# Persistent Nonviral Plasmid Vector in Nasal Tissues Causes False-Positive SARS-CoV-2 Diagnostic Nucleic Acid Tests

**DOI:** 10.1128/spectrum.01695-22

**Published:** 2022-10-13

**Authors:** Ingrid A. Beck, Sheila Styrchak, Leslie Miller, Fred Mast, Vladimir Vigdorovich, Winnie Yeung, Daisy Ko, Alyssa Oldroyd, Samantha Hardy, Song Li, John Houck, Yonghou Jiang, Nicholas Dambrauskas, Catherine Darcey, Andrew Raappana, William Selman, D. Noah Sather, John D. Aitchison, Whitney E. Harrington, Lisa M. Frenkel

**Affiliations:** a Center for Global Infectious Disease Research, Seattle Children’s Research Institute, Seattle, Washington, USA; b Department of Pediatrics, University of Washington, Seattle, Washington, USA; c Department of Global Health, University of Washington, Seattle, Washington, USA; d Department of Biochemistry, University of Washington, Seattle, Washington, USA; e Department of Laboratory Medicine and Pathology, University of Washington, Seattle, Washington, USA; f Department of Medicine, University of Washington, Seattle, Washington, USA; UJF-Grenoble 1, CHU Grenoble

**Keywords:** SARS-CoV-2, plasmid vector, nasal swab, diagnostic test, false test result

## Abstract

Biomedical personnel can become contaminated with nonhazardous reagents used in the laboratory. We describe molecular studies performed on nasal secretions collected longitudinally from asymptomatic laboratory coworkers to determine if they were infected with severe acute respiratory syndrome coronavirus 2 (SARS-CoV-2) circulating in the community or with SARS-CoV-2 DNA from a plasmid vector. Participants enrolled in a prospective study of incident SARS-CoV-2 infection had nasal swabs collected aseptically by study staff at enrollment, followed by weekly self-collection of anterior nasal swabs. SARS-CoV-2 diagnosis was performed by a real-time PCR test targeting the nucleocapsid gene. PCR tests targeting SARS-CoV-2 nonstructural protein 10 (nsp10), nsp14, and envelope and three regions of the plasmid vector were performed to differentiate amplification of SARS-CoV-2 RNA from the plasmid vector’s DNA. Nasal swabs from four asymptomatic coworkers with positive real-time PCR results for the SARS-CoV-2 nucleocapsid targets were negative when tested for SARS-CoV-2 nsp10, nsp14, and envelope protein. However, nucleic acids extracted from these nasal swabs amplified DNA regions of the plasmid vector used by the coworkers, including the ampicillin and neomycin/kanamycin resistance genes, the promoter-nucleocapsid junction, and unique codon-optimized regions. Nasal swabs from these individuals tested positive repeatedly, including during isolation. Longitudinal detection of plasmid DNA with SARS-CoV-2 nucleocapsid in nasal swabs suggests persistence in nasal tissues or colonizing bacteria. Nonviral plasmid vectors, while regarded as safe laboratory reagents, can interfere with molecular diagnostic tests. These reagents should be handled using proper personal protective equipment to prevent contamination of samples or laboratory personnel.

**IMPORTANCE** Asymptomatic laboratory workers who tested positive for SARS-CoV-2 for days to months were found to harbor a laboratory plasmid vector containing SARS-CoV-2 DNA, which they had worked with in the past, in their nasal secretions. While prior studies have documented contamination of research personnel with PCR amplicons, our observation is novel, as these individuals shed the laboratory plasmid over days to months, including during isolation in their homes. This suggests that the plasmid was in their nasal tissues or that bacteria containing the plasmid had colonized their noses. While plasmids are generally safe, our detection of plasmid DNA in the nasal secretions of laboratory workers for weeks after they had stopped working with the plasmid shows the potential for these reagents to interfere with clinical tests and emphasizes that occupational exposures in the preceding months should be considered when interpreting diagnostic clinical tests.

## INTRODUCTION

Human infections with severe acute respiratory syndrome coronavirus 2 (SARS-CoV-2) can be asymptomatic or cause mild, moderate, or severe disease. To determine the biomarkers predictive of these varied outcomes, we conducted a prospective study of incident SARS-CoV-2 infection. Biomedical researchers and/or household members were enrolled, and the study participants self-collected anterior nasal swabs and delivered them to the testing site weekly. Testing of the nasal swabs by PCR detected SARS-CoV-2 in 14 cases within the cohort, including four asymptomatic coworkers and one of their household members who had no known exposures to SARS-CoV-2-infected individuals. When this “outbreak” was noted, epidemiologic investigation revealed that the four coworkers within this research laboratory were using a plasmid vector to express the SARS-CoV-2 nucleocapsid in the month prior to enrollment. In this report, we describe the molecular studies performed to determine if these five case participants were infected with SARS-CoV-2 circulating in the community or with the laboratory plasmid.

## RESULTS

**FIG 1:**
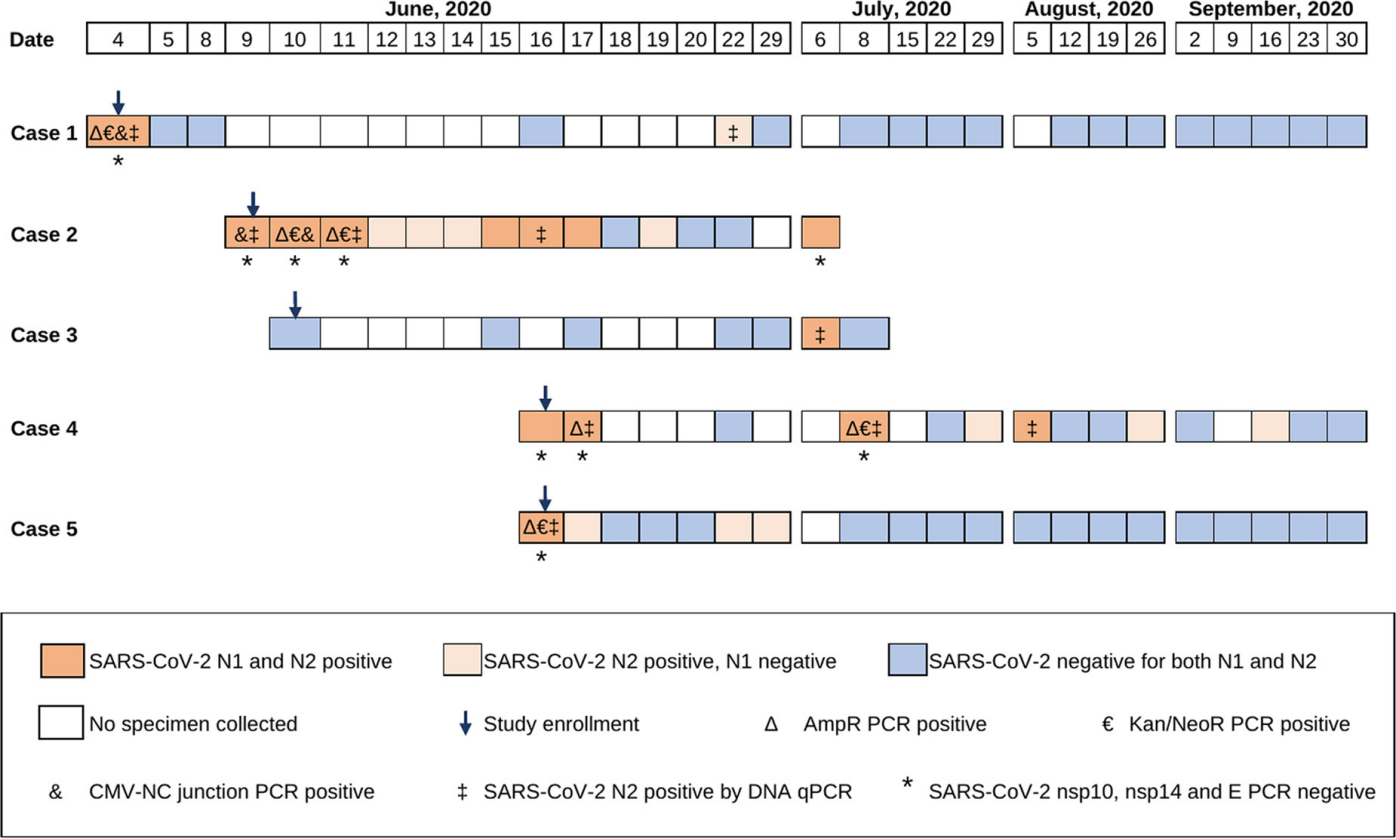
Studies to differentiate SARS-CoV-2 and a vector expressing SARS-CoV-2 nucleocapsid in nasal secretions from five asymptomatic adults. The tests performed on nasal swabs (NS) are graphically depicted across time (dates shown at top). Positive diagnostic real-time PCR tests for the SARS-CoV-2 nucleocapsid gene are indicated by orange rectangles and negative tests by blue rectangles; light orange indicates that only the N2 region was amplified, often at very low levels (*C_T_*, 40 to 44), and dark orange indicates that both the N1 and N2 regions were amplified (N2 *C_T_*, 33.2 to 42.2). Nucleic acids from the NS with the lowest threshold cycle values (i.e., the highest viral loads) in the SARS-CoV-2 diagnostic assay were tested for additional regions of SARS-CoV-2 outside the nucleocapsid and for regions of the vector (pcDNA 3.4 TOPO; Life Technologies, CA). nsp10 and nsp14, nonstructural proteins 10 and 14; E, envelope; AmpR, ampicillin resistance; Kan/NeoR, kanamycin/neomycin resistance; CMV-NC junction, CMV promoter-SARS-CoV-2 nucleocapsid junction, confirmed by sequencing.

Nasal swabs collected between 4 and 16 June 2020 from four coworkers at the research laboratory (Fig. 1; cases 1, 2, 4, and 5) tested positive for SARS-CoV-2 at study enrollment, and one of their household members tested positive 3 weeks after enrollment (Fig. 1; case 3). Additional follow-up nasal swabs from the four coworkers also tested positive on subsequent dates (Fig. 1). The household member had a single nasal swab positive for SARS-CoV-2, followed by a negative swab collected 2 days later before being lost to follow-up. The median real-time PCR cycle threshold (*C_T_*) values for N1 and N2 in the positive nasal swabs were 38.5 (range, 32.5 to 42.8) and 39.3 (range, 33.2 to 44.1), respectively. A total of 14 specimens were positive for both N1 and N2, and 11 additional specimens had only N2 detected at *C_T_* values between 38.8 and 44.1 (Table 1). The coworkers had no known exposures to pandemic SARS-CoV-2. All subjects underwent 10 days of isolation in their homes and had home visits, with specimens collected aseptically by study personnel. The study participants remained asymptomatic throughout follow-up. SARS-CoV-2 antibodies were not detected in plasma from enrollment in any of the five cases or after 4 weeks of follow-up in the four coworkers. The household member did not have follow-up plasma available for antibody testing.

**TABLE 1:** Real-time PCR diagnostic test results detecting SARS-CoV-2 nucleocapsid in nasal specimens with and without the reverse transcription step^*a*^

Case no.	Visit type	Sample date (mo/day/yr)	*C_T_* for diagnostic:		
			RT-PCR		PCR
			N1	N2	N2
1	ENR	6/4/2020	33.7	34.5	36.0
	FU	6/22/2020	NA	40.1	38.7
2	ENR	6/9/2020	38.9	36.1	36.7
	CONF	6/10/2020	32.9	33.2	NT
	FU	6/11/2020	40.6	36.3	38.9
	FU	6/12/2020	NA	38.8	NT
	FU	6/13/2020	NA	41.4	NT
	FU	6/14/2020	NA	40.6	NT
	FU	6/15/2020	41.5	38.1	NT
	FU	6/16/2020	40.4	40.1	39.9
	FU	6/17/2020	36.3	39.2	NT
	FU	6/19/2020	NA	41.9	NT
	FU	7/6/2020	36.6	37.1	NT
3	FU	7/6/2020	39.3	42.4	38.9
4	ENR	6/16/2020	38.5	36.5	NT
	CONF	6/17/2020	32.5	33.8	38.9^*b*^
	FU	7/8/2020	35.2	35.3	37.0^*b*^
	FU	7/29/2020	NT	42.8	NT
	FU	8/5/2020	42.8	42.2	37.6
	FU	8/26/2020	NT	41.6	NT
	FU	9/16/2020	NA	44.1	NT
5	ENR	6/16/2020	38.4	38.3	37.9
	CONF	6/17/2020	NA	39.3	NT
	FU	6/22/2020	NA	42.3	NT
	FU	6/29/2020	NT	41.4	NT

a RT-PCR, reverse transcription-PCR; N1, nucleocapsid target 1; N2, nucleocapsid target 2; *C_T_*, cycle threshold; ENR, enrollment; FU, follow-up; CONF, confirmation; NA, no amplification detected; NT, not tested.

b Test results in all three columns are from the same aliquot of extracted nucleic acid, except in two samples with insufficient nucleic acid remaining, which had a separate aliquot from the original nasal swab specimen extracted and tested; the second aliquot RT-PCR N2 Ct values (not shown in the table) were 38.1 (sample from 17 June 2020) and 36.2 (sample from 8 July 2020).

Additional testing of the five cases’ N1- and N2-positive nasal swabs for regions of SARS-CoV-2 RNA outside the nucleocapsid, including nonstructural protein 10 (nsp10), nsp14, and envelope (E), were negative. However, multiple PCR assays conducted using nucleic acids without reverse transcription from the cases’ specimens amplified DNA sequences unique to the plasmid used in the coworkers’ laboratory. Specifically, the ampicillin and kanamycin/neomycin resistance genes were amplified from four of the five cases, and the cytomegalovirus (CMV) promoter-nucleocapsid junction, which included a codon-optimized synthetic sequence unique to this plasmid construct, was amplified from two of the five cases (Fig. 1). The latter amplicons were confirmed by sequencing to be identical to the laboratory plasmid. Additionally, repeat testing of these nasal swabs for SARS-CoV-2 nucleocapsid using the diagnostic assay (except with omission of the reverse transcription step) amplified the N2 region, with *C_T_* values similar to those of the original real-time reverse transcription-PCR (RT-PCR) of all cases (Table 1), indicating amplification of SARS-CoV-2 nucleocapsid DNA, presumably from plasmid rather than viral RNA.

## DISCUSSION

SARS-CoV-2 was amplified from longitudinally collected nasal specimens from five asymptomatic individuals, four coworkers and one of their household members, which suggested person-to-person transmission of pandemic coronavirus. However, after the study team learned that a plasmid vector had been used in the month prior to enrollment by all four case coworkers in the laboratory outbreak, additional studies were performed to differentiate SARS-CoV-2 RNA from virus circulating in the community from DNA from the laboratory plasmid. Our inability to amplify regions of SARS-CoV-2 RNA outside the nucleocapsid that serve as targets in other diagnostic assays (1) did not support the presence of the pandemic SARS-CoV-2 in these nasal swabs. Multiple molecular assays detected DNA sequences, including a codon-optimized region unique to the plasmid used by this research group to produce SARS-CoV-2 nucleocapsid protein, in each of the case coworker’s nasal specimens (Fig. 1). Taken together, these results indicate that the plasmid DNA encoding the SARS-CoV-2 nucleocapsid gave false-positive results in our SARS-CoV-2 diagnostic assay.

Unlike prior reports of false diagnostic test results due to contamination of specimens by PCR amplicons (2, 3), the nasal specimens from the individuals we studied remained positive over days to weeks, including when the individuals were in isolation in their homes. The prolonged detection of SARS-CoV-2 nucleocapsid plasmid DNA from longitudinally collected nasal swabs suggests that the plasmid was present in their nasal tissues or that the Escherichia coli containing the plasmid had colonized their noses.

The coworkers in our study used laboratory protocols to amplify and purify plasmids that included handling high-titer bacterial cultures and conducting procedures with the potential for generating aerosols (pouring liquids, centrifugation, removing supernatant and resuspending pellets, vortexing, etc.), which may pose a risk of inhalation. Bacterial strains and reagents used for cloning are generally not considered to be infectious materials. Therefore, most of these procedures are performed on the “open bench” in biosafety level 2 (BSL2) laboratories, following standard laboratory practices and using proper personal protective equipment (PPE), including gloves, lab coats, goggles, and safety centrifuge cups. Additionally, solutions of the purified plasmid are highly concentrated and can contaminate gloves, equipment, and the research environment, similarly to surface contamination with PCR amplicons (4). Several studies have shown high stability of plasmid DNA under various conditions (5, 6) and persistence of DNA on surfaces even after decontamination (4).

The nonviral SARS-CoV-2 DNA from the nasal specimens collected longitudinally from the coworker cases was amplified over weeks to months, and the household member who did not work in a laboratory had a single positive test (this individual was only sampled once following their positive nasal specimen), illustrating the potential for this type of reagent to be carried from the lab to the home. While the initial positive swabs were collected aseptically by study personnel, we cannot rule out whether these or subsequent self-collected nasal swabs were contaminated by plasmid-contaminated hands or surfaces. However, laboratory work with the plasmid encoding the SARS-CoV-2 nucleocapsid was performed between mid-April and 3 June 2020 and completed before the coworkers enrolled in the study. Additionally, the enrollment nasal swabs were collected by the clinical team in a dedicated health room away from the laboratories, and confirmatory samples were also collected by study staff at the participants’ homes while they were in isolation, which suggests that the plasmid persisted within the nasal cavity for days to weeks.

Plasmid vectors, commonly used in research, can contaminate laboratory reagents (7) and biological samples (8), leading to false scientific claims (8, 9) or diagnostic results (2–4), which can create emotional stress for patients and divert health care resources to further tests. While plasmids are regarded as safe laboratory reagents, personnel handling plasmid solutions should use PPE and dedicated laboratory areas physically separated from spaces used for sample collection, processing, or nucleic acid amplification. Our novel documentation of plasmid DNA in nasal secretions for weeks following exposure highlights the potential for these reagents to interfere with molecular diagnostic tests performed on research personnel and emphasizes that occupational exposures occurring weeks to months in the past should be considered when interpreting diagnostic clinical tests.

## MATERIALS AND METHODS

### Study design and setting.

The SARS-CoV-2 prospective cohort study (SARS2 Study) performed at the Seattle Children’s Research Institute enrolled participants between 3 April and 23 July 2020. Following informed consent, as approved by the Seattle Children’s Institutional Review Board, Seattle Children’s and University of Washington employees and their household members were screened for SARS-CoV-2 exposures and symptoms and risk factors associated with COVID-19 disease severity and had blood and nasal swabs collected aseptically by the study personnel. Subsequently, each participant completed a weekly survey reporting contacts with others and respiratory and other symptoms and provided a self-collected nasal swab sampling both anterior nasal cavities for SARS-CoV-2 testing over the course of 54 weeks. Participants testing positive for SARS-CoV-2 (cases) had home visits by the study team to collect nasal specimens to confirm infection and to document their vital signs and oximetry. After detection of SARS-CoV-2, these “case participants” collected nasal swabs twice weekly until testing negative on two sequential specimens, then resumed weekly collection.

### SARS-CoV-2 diagnostic assay.

SARS-CoV-2 diagnosis was performed using a laboratory-developed real-time PCR assay with Emergency Use Authorization from the Washington Department of Health. The assay amplified two regions of the SARS-CoV-2 nucleocapsid gene (N1 and N2) (10), following reverse transcription of the RNA (iTaq universal probes one-step kit; Bio-Rad Laboratories, CA). The real-time PCR was run for 45 cycles, and the diagnostic cutoff for SARS-CoV-2 positivity was set at a cycle threshold (*C_T_*) of 40. Processing of the nasal swabs and diagnostic testing were performed in a Clinical Laboratory Improvement Amendments (CLIA)-certified laboratory.

### Tests to differentiate SARS-CoV-2 and a vector expressing SARS-CoV-2 nucleocapsid.

To determine if nasal swabs from the five cases indeed contained SARS-CoV-2 RNA and/or the plasmid, nucleic acids extracted from the nasal swabs were subjected to additional testing. These included reverse transcription and PCR to amplify the SARS-CoV-2 RNA encoding nonstructural protein 10 (nsp10), nsp14, and envelope (E) (1). In addition, PCR was performed to amplify regions of the vector DNA (pcDNA 3.4 TOPO; Life Technologies), including genes for ampicillin and kanamycin/neomycin resistance and the CMV promoter-SARS-CoV-2 nucleocapsid junction.

### SARS-CoV-2 antibody testing.

Plasma samples collected at study enrollment, at home visits a few days after the initial detection of SARS-CoV-2, at 21 and 28 days following detection, and at the final study visit were tested for seroconversion to SARS-CoV-2 spike protein using the SCoV-2 Detect IgG and IgM enzyme-linked immunosorbent assay (ELISA; InBios, Seattle, WA).
